# Review on *"Atkins Diabetes Revolution: The Groundbreaking Approach to Preventing and Controlling Type 2 Diabetes" *by Mary C. Vernon and Jacqueline A. Eberstein

**DOI:** 10.1186/1743-7075-1-14

**Published:** 2004-11-09

**Authors:** Surender Arora, Samy I McFarlane

**Affiliations:** 1 State University of New York Downstate Medical Center, 450 Clarkson Avenue, Brooklyn, New York 11203, USA; 2 Kings County Hospital, Brooklyn, New York, USA

Before beginning the review of this book, we had no particular opinion about the role of low carbohydrate diets in diabetes. In order to write a fair and unbiased review, we have done a rather extensive search on the subject. One of the most disturbing findings of our search is the amount of hostility towards low carbohydrate diets that is on the web and in the scientific literature. We found several sites that present no scientific arguments but are, rather, full of *ad hominem *attacks. This was particularly disturbing in that we are in the midst of a growing epidemic of obesity and diabetes with very alarming figures and projections from all over the world. Any intervention that has the potential for helping curb this dangerous epidemic which claims thousands of lives every day should be looked at with a great deal of objectivity.

The low carbohydrate approach, in fact, is not new and was used in England more than a century ago, made popular by William Harvey [1], an ENT surgeon. He prescribed a low carbohydrate diet for William Banting, an obese carpenter who had been having a great difficulty losing weight. Banting was able to lose weight and as a service, he published in 1863 a small booklet entitled *Letter on Corpulence Addressed to the Public *[2], the first book to be published on obesity and one which popularized low carbohydrate diets. He has been called "Father of low carbohydrate diets" and was honored by his name being included in the dictionary as the verb "to bant" meaning "to diet". The low carbohydrate diet also been called a "Harvey-Banting diet" after the names of these pioneer. Since then, it has been in and out of fashion with different versions and names but with the same underlying concept, most recently popularized by the late Dr. Robert C. Atkins.

The Atkins Diabetes Revolution [3] plan is similar to the Atkins weight loss strategy: four levels of carbohydrate restriction are instituted. The induction phase restricts dieters to 20 g of carbohydrate. On the weight loss plan, this is recommended for about 2 weeks. In diabetes this is maintained until glycemic control is attained. In the latter stages, carbohydrates are added as long as weight loss or stability is maintained. For diabetes, carbohydrates are only reintroduced if glycemic control is acceptable. In the later phases, the Atkins Diabetes plan adds a Glycemic Ranking (AGR), derived from the glycemic index, glycemic load and net carbs. Preference is given to whole fruits and berries and juices and dried fruits are low on the list. As in weight loss, exercise is "mandatory."

The Atkins Diabetes Revolution book is an attempt by the authors to present the low carbohydrate diet as a preventive and treatment strategy for patients with type 2 diabetes and those with the metabolic syndrome, who are at high risk for developing diabetes and cardiovascular disease. In doing so, the book, which is very well written, and which clearly presents illustrative cases, explains very complex metabolic concept in a very easy to read and understandable format. The first nine chapters explain the different concepts involved in glucose and lipid metabolism and the interplay of the various cardiovascular risk factors that culminate in cardiovascular disease the number one killer of Americans today. Definitions of metabolic syndrome, pre-diabetes, body mass index, waist to hip ratio, central obesity and their relationship to diabetes, heart attacks and strokes, are eloquently presented with a great deal of accuracy yet in a simple format. Most impressive were the case presentations, especially that of reactive hypoglycemia and carbohydrate craving. This response is associated with hyperinsulinemia in the pre-diabetic phase and sometimes puzzles clinicians unless they know to look for it.

The second section of the book is devoted to an in-depth discussion of the various macro and micronutrients and their role in diabetes and obesity. Concepts such as the glycemic index and glycemic load are very well illustrated. The last section consists of meal plans and menus of low carbohydrate diet that the book is advocating.

The concept of low carbohydrate diet and glycemic control certainly has a pathophysiological merit. First, dietary carbohydrates are the principal source for the initial rise of glucose in the diabetic populations, who generally have a defect in the first phase insulin secretion that is responsible for handling the glucose load [4]. There is mounting evidence that postprandial hyperglycemia is in itself a risk factor for cardiovascular disease in the diabetic patients [5]. This evidence comes from large, well-conducted, randomized controlled trials [5,6]. Furthermore, control of postprandial hyperglycemia has been shown to provide cardiovascular benefits, and contribute to the overall decrease of hemoglobin A_1c_, something that has been clearly shown to reduce microvascular disease in both type 1 and type 2 diabetes [7,8]. Second, the initial blood glucose rise associated with high carbohydrate load, in the presence of absolute/relative insulin deficiency leads to significant rise in triglycerides and free fatty acids which perpetuate the cycle of insulin resistance [9,10]. So, from a metabolic stand point, low carbohydrate diet makes physiologic sense. However, in the science and practice of medicine, not everything that makes sense turns out to work the way it is supposed to. In looking at the low carbohydrate diet, we must examine the evidence from the studies that were conducted using such diets keeping in mind that weight loss by itself, is beneficial in terms of improving insulin sensitivity and correcting the abnormalities associated with the metabolic syndrome and insulin resistance [9,10]. Also, weight loss has much greater effect on the prevention of type 2 diabetes in pre-diabetic patients than pharmacological interventions [9]. This fact was well illustrated in the Diabetes Prevention Program, a large multicenter trial sponsored by the National Institute of Health, where pre-diabetic patients on diet and exercise program had a 58% reduction in the development of diabetes, compared to only 34% reduction with the use of metformin [11]. This landmark study had a population where women and minorities were very well represented [11]. The fact that weight loss was associated with reduction of type 2 diabetes in high risk populations was illustrated in several other studies including examples from Finland and from China, making it evident that weight loss works for a variety of ethnic populations [12-15].

In two recent randomized controlled trials published in the New England Journal of Medicine [16,17], the effects of low carbohydrate and low fat diets were compared in obese and diabetic patients. Both of these studies showed a substantial decrease of triglycerides in patients on low carbohydrate diet with simultaneous increase in high-density lipoprotein (HDL) over 6 month to 1 year period. The studies did not show a change in the low-density lipoprotein (LDL) values in the low carbohydrate group compared to their baseline, while those on traditional low fat diet had a reduction in LDL levels. Patients on low carbohydrate diet, however, had substantially significant weight loss, almost double that achieved with the traditional diet, in the first 3–6 months. At one year, there was no significant difference in weight loss between the two groups [16-18]. Although participants on the low carbohydrate diet initially tended to have higher rate of side effects such as nausea, muscle cramps and constipation, compliance with diet was similar in both groups. In fact, more participants adhered to the low carbohydrate diet. Although weight loss was similar after one year between groups, the effects on atherogenic dyslipidemia and glycemic control were still more favorable with a low-carbohydrate diet after adjustment for differences in weight loss.

Despite the evidence from these randomized controlled trials, published in the prestigious New England Journal of Medicine, there is a significant amount of reluctance in the scientific community to acknowledge the beneficial effects of low carbohydrate diets. These studies, in fact, provide a striking example of this resistance. A commentary in the same issue of the New England Journal of Medicine [20] states that "In both studies, the reduction in serum triglyceride levels in subjects randomly assigned to the low-carbohydrate diet might have been anticipated as a result of their greater weight loss, although it is true that reduced carbohydrate intake is generally associated with reduced triglyceride levels" [20]. In this statement, despite the fact that low carbohydrate diet is known to reduce serum triglyceride, the authors suggest otherwise. In another statement, the authors of the commentary state that "the rise in HDL cholesterol in the subjects following the low-carbohydrate diet (a change observed only by Foster et al.) may reflect a change in HDL subfractions that occurs with increased intake of saturated fats, and this change has not been shown to be beneficial. Thus, caution is urged about over-interpretation of this observation as a beneficial result of a low-carbohydrate, high-fat diet" [20]. Again this statement illustrates the difficulty in acknowledging what a randomized controlled trial has shown. The authors suggest, without any evidence that the rise in HDL cholesterol might have been in the non-beneficial HDL subfraction. In other words, when low carbohydrate diet is shown to decrease triglycerides, a suggestion is made that it might be just secondary to weight loss and when this diet increases HDL, it is also suggested that it could be the non-beneficial HDL. Now, let us examine the evidence provided by the one year follow-up study on the same group of patients where the investigators conclude that "Although weight loss was similar between groups, the effects on atherogenic dyslipidemia and glycemic control were still more favorable with a low-carbohydrate diet after adjustment for differences in weight loss" [18]. This indicates that the statements made in the commentary [20], in an attempt to dismiss or downplay the beneficial effects of low carbohydrate diet were simply wrong. Furthermore, the statement made in the commentary regarding the HDL cholesterol, not only lacks objective evidence, but also contradicts the current findings that lowering insulin level by controlled carbohydrates shift HDL production to a much more desirable, lighter HDL_2_subfractions [21,22].

On the other hand, the American Diabetes Association, despite recommending the traditional low fat diet, has recently reduced the recommended carbohydrate contents in the diet, perhaps reflecting a trend towards a reduced carbohydrate diet to follow [19].

Returning to the Atkins book, despite the fact that the book is very well referenced, certain statements such as "high carbohydrate diet leads to diabetes" are not well substantiated, unless of course such a diet leads to weight gain, which it may. Furthermore, the book does not devote a sufficient amount of space discussing the side effects associated with dieting in general and low carbohydrate diet in particular. This is of concern, since it leaves the reader with the impression that the low carbohydrate diet or dieting, in general, has no negative consequences. Nonetheless, the amount of information the book provides in a simple, yet accurate format will benefit patients with diabetes and their families as well as those who are at risk for developing diabetes and the metabolic syndrome. If, after reading this book, the reader is able to identify that he or she is at risk for diabetes and the metabolic syndrome and takes action that could potentially save his or her life the book will be a valuable contribution. ***Atkins Diabetes Revolution ***has a list price of $25.95 and is available at Amazon.com and presumably other sites for half that price. Possibly, a shorter and still more affordable version of the book would be helpful for diabetic patients, their families and for the general reader, to help identify their risk for the disease.

As clinicians, we would not be comfortable recommending any diet without first hand experience. The ***Atkins Diabetes Revolution***, however, is sufficiently convincing to make us believe that some form of low carbohydrate intervention is worth investigating and should be considered by practitioners. The highly negative un-scientific response of critics, if anything, encourages us in this direction.
